# Supplementary material for: Process of Glucose Increases Rather Than Constant High Glucose Was the Main Cause of Abnormal Glucose Induced Glomerulus Epithelial Cells Inflammatory Response
Source: Int J Mol Sci. 2022 Dec 29;24(1):600. doi: 10.3390/ijms24010600 (PMC9820529; doi:10.3390/ijms24010600)
Supplement: Supplementary file 1 [file ijms-24-00600-s001.zip › ijms-2065129-supplementary.pdf]

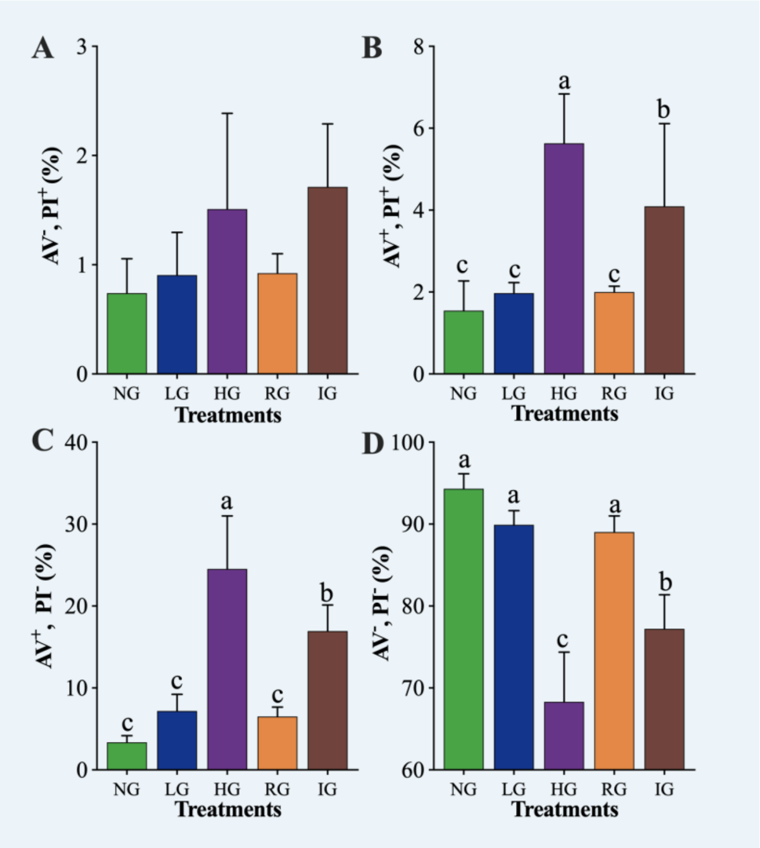

Supplementary Figure S1. The flow cytometry results of the Annexin V-FITC/PI assay are involved in Figure 1 (n=3).

Supplementary Table S1. The primers used in the RT-qPCR experiments

Gene Name	Primer sequence (5'-3')
nox4	Forward: GCTTTGGATTTCTGGACCTTTGTGC<br>Reverse: CTGACGGATGACTTGTGACTGAGATG
p2x4	Forward: TGAGGGAGGCATCATGGGCATC<br>Reverse: TCTAGGCGGCGGAAGGAATACC
txnip	Forward: AGCCAGCCAACTCAAGAGACAAAG<br>Reverse: AGACAGACACCCGCCCATCAG
nlrp3	Forward: AAGAAGAGGAGGAAGTTA<br>Reverse: GTCAGATAGTTCACCAAT
caspase-1	Forward: CTGAACCAGGAGGAGATG<br>Reverse: TTACGAATAACAGTGTCTATCAA
gsdmd	Forward: AAGGTGGTCAGGAGCGTCGTC<br>Reverse: CGGTCTCCAGAATCGTGAACTCAAG
β-actin	Forward: CGTCCGTGACATCAAGGAGAAGC<br>Reverse: GGAACCGCTCATTGCCGATGG
